# Cod-Liver Oil Improves Metabolic Indices and hs-CRP Levels in Gestational Diabetes Mellitus Patients: A Double-Blind Randomized Controlled Trial

**DOI:** 10.1155/2019/7074042

**Published:** 2019-12-28

**Authors:** Shuli Yang, Ruixin Lin, Lihui Si, Zhuo Li, Wenwen Jian, Qing Yu, Yan Jia

**Affiliations:** ^1^Department of Gynecology and Obstetrics, The Second Hospital of Jilin University, Changchun 130041, China; ^2^Department of Hepatopancreatobiliary Surgery, The Second Hospital of Jilin University, Changchun 130041, China

## Abstract

**Objective:**

To investigate the effects of cod-liver oil on metabolic status and high-sensitivity C-reactive protein (hs-CRP) in patients with gestational diabetes mellitus (GDM).

**Methods:**

This study was a randomized, double-blinded, placebo-controlled trial with the allocation ratio of 1 : 1. The contents of EPA and DHA in cod-liver oil were measured using a gas chromatograph. A total of 550 GDM patients were randomly divided into the intervention group (cod-liver oil) and the control group (placebo, mineral oil), and both groups were given regular dietary care. Glycosylated hemoglobin A1c (HbA1c), fasting plasma glucose (FPG), 2-hour plasma glucose (2hPG), lipid profiles, homeostatic model assessment insulin resistance (HOMA-IR), and hs-CRP were measured. Primary outcomes were different in HbA1c, FPG, 2hPG, and HOMA-IR between the two groups after 4-week randomization. Secondary outcomes were the blood glucose levels and perinatal complications (pregnancy-induced hypertension, polyhydramnios, premature delivery, postpartum hemorrhage, postpartum infection, premature rupture of membranes, and cesarean section) between the two groups before and after 12-16 weeks of cod-liver oil intervention from middle pregnancy to late pregnancy.

**Results:**

EPA and DHA were the main components of cod-liver oil with 76 mg/mL and 150 mg/mL, respectively. There was no significant difference for primary outcomes in the levels of HbA1c, FPG, 2hPG, HOMA-IR, and lipid profiles between the two groups (*P* > 0.05). For the secondary outcomes, the levels of HbA1c, FPG, 2hPG, triglyceride (TG), total cholesterol (TC), low-density lipoprotein cholesterol ratio (LDL-C), HOMA-IR, and hs-CRP in the intervention group were significantly lower than those in the control group (*P* < 0.05). The incidence of perinatal complications in the intervention group was lower than that in the control group too (*P* < 0.05).

**Conclusions:**

Cod-liver oil consumption effectively reduced the levels of blood glucose, lipid levels, hs-CRP, and HOMA-IR and the incidence of perinatal complications.

## 1. Introduction

Gestational diabetes mellitus (GDM) is a disease that occurs during pregnancy. GDM affects 9.8%–25.5% of pregnant women in the world, and the GDM incidence rate was approximately 17.5% in China [1]. It is caused by varying degrees of impaired glucose tolerance [2] and different degrees of hyperglycemia caused by diabetes [3]. At present, the diagnosis criteria of GDM are still not unified, so the incidence of GDM reported is quite different in the literature. However, with the improvement of people's living standards and changes in dietary structure, the incidence of GDM in China has increased significantly [4]. GDM can have a great impact on the health of the mother and the fetus, leading to adverse pregnancy outcomes. Dietary intervention can effectively control the blood glucose level of patients and reduce the occurrence of perinatal maternal and child complications [5].

Omega-3 fatty acids are involved in human physiology, including *α*-linolenic acid (ALA) [6, 7] and eicosapentaenoic acid (EPA) and docosahexaenoic acid (DHA) in cod-liver oil [8]. Omega-3 fatty acids were found to reduce fasting plasma glucose (FPG), homeostatic model of assessment for insulin resistance (HOMA-IR), and high-sensitivity C-reactive protein (hs-CRP) in the women with gestational diabetes [9]. EPA and DHA are important omega-3 fatty acids that are indispensable to the human health [10] but the human body cannot produce them directly [11]. They have various physiological effects such as lowering blood lipids, blood pressure, and cholesterol, preventing arteriosclerosis [12], reducing thrombosis [13], and preventing cardiovascular [14] and cerebrovascular diseases [15]. In addition to the above effects, DHA can protect the retina, improve vision [16], promote infants' intellectual development, and improve memory [17]. The recommended mean intake for EPA and DHA was 100 mg/d in USA, which is much lower than what many groups worldwide are recommended [18], and a consensus document recommended 200 mg/d of DHA for pregnant women [19]. The recommended values are also used in Chinese pregnant women [20].

However, EPA and DHA are difficult to measure directly. The fatty acid methyl esterification is often considered for the measurement of EPA and DHA content in fish oil via potassium hydroxide transesterification [21], methyl esterification [22], boron trifluoride methylation [23], and sulfate methylation [24]. The above-mentioned literature was used to improve the determination of fatty acids in cod-liver oil. The gas chromatography (GC) detection and the derivatization reaction conditions were optimized, and the GC internal standard method was established to rapidly determine the contents of EPA and DHA in cod-liver oil. In this study, 550 patients with GDM were enrolled in this study. FPG, 2hPG, HOMA-IR and hs-CRP and perinatal complications were investigated after cod-liver oil consumption, so as to explore dietary control for gestational diabetes.

## 2. Materials and Methods

### 2.1. Reagents

Isooctane and methanol (chromatographically pure) were purchased from JT Baker (Phillipsburg, NJ, USA). Sodium hydroxide, sulfuric acid, and anhydrous sodium sulfate (analytical grade) were purchased from Sinopharm Chemical Reagent Co. Ltd. (Beijing, China); EPA methyl ester (batch number, E6540099), DHA methyl ester (batch number, I2920075), EPA (batch number, H0660050), and DHA (batch number, I2950035) were provided by Shanghai Anpu Experimental Technology Co., Ltd.(Shanghai, China); and tetracosanoic acid (C23:0, purity ≥ 99.0%) and methyl ester (batch number, BCBV4775; purity ≥ 99.0%) were purchased from Sigma. Bet's Xiaofu cod-liver oil (in capsule form) was purchased from Guangdong Meikang Health Products Co., Ltd. (Guangzhou, China). The approved document no. was Guojian G20130699 by the State Food and Drug Administration.

### 2.2. Solution Preparation

Fifty mg of each C23:0 methyl ester, EPA methyl ester, and DHA methyl ester was mixed in a 10 mL brown volumetric flask, dissolved with isooctane, and diluted to 10 mL. Two hundred fifty mg of C23:0 methyl ester was placed in a 50 mL brown volumetric flask, dissolved with isooctane, and diluted to 50 mL. Four hundred mg of each EPA methyl ester and DHA methyl ester was placed in a 20 mL brown volumetric flask, dissolved with isooctane, and diluted to 20 mL. On hundred *μ*L of cod-liver oil was placed in a 20 mL screw-on reaction flask, and 1.5 mL of 2% sodium hydroxide in methanol solution was added. The solution was vortexed for 30 s, heated in a 90°C thermostat for 20 min, and cooled to room temperature. Two mL of 5% sulfuric acid-methanol solution was added, mixed for 30 s, heated in a 100°C thermostat for 10 min, and cooled to room temperature. The aspirated supernatant was transferred to a test tube containing a small amount of anhydrous sodium sulfate and shaken for dehydration.

### 2.3. Gas Chromatography (GC) Analysis of EPA and DHA

An Agilent 7890A Gas Chromatograph was purchased from Agilent (Folsom, CA, USA) with a DB-23 Capillary Column (30 m × 0.25 mm, 25 *μ*m). The following HPLC condition was used: starting temperature was 170°C, ramping up to 225°C at 1°C/min and holding for 5 min, inlet temperature was 250°C, detector temperature was 280°C, carrier gas was high purity N_2_, flow rate was 1.0 mL/min, injection volume was 1 *μ*L, injection method was split injection, and split ratio was 100 : 1. The mixed reference solution was taken and injected under the above chromatographic conditions. The two solutions were each continuously injected with 5 needles, and the peak area was recorded and the relative correction factor (*F*_*X*_) was calculated. The results are shown in Table 1.

### 2.4. Standard Curve Preparation

Different volumes of stock solution were taken in 0, 0.05, 0.5, 1.0, 2.0, and 10 mL and placed in a 20 mL volumetric flask. 2 mL of the internal standard solution was added and diluted with isooctane to make the EPA concentration of 0.049, 0.487, 0.974, 1.948, 4.870, and 9.740 mg/mL, respectively; a series of DHA were prepared at the concentrations of 0.0510, 0.510, 1.021, 2.041, 4.082, and 10.206 mg/mL, respectively. According to the above chromatographic conditions, the peak area was recorded. The EPA and DHA methyl ester concentration/internal standard concentration ratios were plotted on the abscissa (*X*), and the EPA and DHA methyl ester peak area/internal standard peak area ratio was the ordinate (*Y*). The EPA methyl ester regression equation was *Y* = 0.9717*X* + 0.0018, *r* = 1.0000; the DHA methyl ester regression equation was *Y* = 0.9629*X* + 0.0008, *r* = 1.0000. The results showed that EPA and DHA showed a good linear relationship between the peak area ratio and concentration ratio in 0.0487~9.740 mg/mL and 0.0510~10.206 mg/mL.

### 2.5. Quantitative Limit and Detection Limit

EPA and DHA solution was taken from a 2 mL or 4 mL volumetric flask and diluted with isooctane, and a solution containing EPA 0.0233 mg/mL and DHA 0.0244 mg/mL was prepared. The sample was measured and the noise ratio (S/N) was 10.32 and 9.66, respectively. The quantitative limits of EPA and DHA were 0.466 mg/g and 0.488 mg/g, respectively. The low concentration of EPA and DHA was drawn from the 1.5 mL or 10 mL volumetric flask and diluted with isooctane, and a solution containing EPA 0.0070 mg/mL and DHA 0.0073 mg/mL was prepared. The signal-to-noise (S/N) ratio of EPA and DHA was 3.25 and 2.92, respectively. The detection limits of EPA and DHA were 0.140 mg/g and 0.146 mg/g, respectively.

### 2.6. Repeatability Test

The same batch of samples (batch number: 170420) was divided into 6 parts and prepared according to the above chromatographic conditions. The peak area was recorded, and the contents of EPA and DHA were calculated. The EPA content was 73.22 mg/g and the RSD was 0.66%; the DHA content was 145.62 mg/g and the RSD was 0.78%, indicating that the method was reproducible.

### 2.7. Detection of the Stability of EPA and DHA

The same sample was placed at room temperature and tested according to the above chromatographic conditions at 0, 4, 8, 12, 16, 20, and 24 h, respectively. The peak area RSD of EPA and DHA methyl ester was 1.67% and 1.89%, respectively, indicating that the sample was stable within 24 h.

### 2.8. Measurement of Recovery Rate of EPA and DHA

Seventy mg of EPA and 140 mg of DHA were placed in a 10 mL volumetric flask to make a mixture containing approximately EPA 7 mg/mL and DHA 14 mg/mL. The sample was divided into 9 parts. The above standard solution of 0.48, 0.6, and 72 mL (each 3 parts) was used to make the loading level of 80%, 100%, and 120%, respectively.  Table 2 showed that the recovery rate of added standards was more than 98%.  Table 3 showed that the contents of n-3 polyunsaturated fatty acids (PUFA) from different batches were stable.

### 2.9. Participants

Before the experiment, all procedures were approved by the Human Research Committee of The Second Hospital of Jilin University. From March to April 2018, 1238 patients visited our hospital and were suspected with GDM at early pregnancy. The women received an oral glucose tolerance test and had more than normal values (fasting level < 5, 1 h level < 10, 2 h level < 7.8 mmol/l). They were collected for further selection in the present study. All patients were singleton pregnancies.

### 2.10. Evaluation of Dietary Intake

A validated semiquantitative food frequency questionnaire (FFQ) table was used to retrospectively investigate the diet of pregnant women during early pregnancy, middle pregnancy, and late pregnancy. The diet included 11 types of 33 foods: cereals (rice, wheat flour, and miscellaneous grains), meat (pork, beef, lamb, chicken/duck, freshwater fish, marine fish, and shrimp/crab/shellfish), milk (fresh milk, milk powder, and yogurt), eggs, soy products (soybean milk, tofu, and other soy products), green leafy vegetables (green vegetables, etc.), other vegetables (cabbage/Chinese cabbage, celery, green beans/beans, carrots, tomatoes, eggplant, potatoes, mushrooms, green/red pepper, bamboo shoot class, fungus, and garlic), seaweed/kelp, pickles/kimchi, dried fruits, and fruits. Food frequency was divided into never eat, daily times, weekly times, and monthly. The final survey results were uniformly converted to daily intake for analysis.

### 2.11. Diagnostic Criteria and Definitions

The stages of pregnancy were divided into early pregnancy (gestational age 8-12 weeks), middle pregnancy (gestational age 13-27 weeks), and late pregnancy (28 to 40 weeks of gestational age 28-40 weeks). Diagnostic criteria for gestational diabetes referred to the guidelines for prevention and treatment of diabetes in China (2014): pregnant women were given an oral glucose tolerance test (OGTT) at 24 to 28 weeks of gestation. The diagnostic criteria were fasting ≥ 5.1 mmol/L and 2 h ≥ 8.5 mmol/L, and the blood glucose level reached or exceeded a certain standard to diagnose GDM. Prepregnancy BMI group: BMI < 18.5 kg/m^2^ for the underweight group, 18.5 kg/m^2^ ≤ BMI < 24 kg/m^2^ for the normal weight group, and BMI ≥ 24 kg/m^2^ overweight/obese group. Weight gain during pregnancy = weight before delivery (kg) − prepregnancy weight (kg). According to the Institute of Medicine (IOM), the range of recommended weight gain during pregnancy for different prepregnancy BMI is divided into three groups: insufficient weight gain during pregnancy, appropriate, and excessive. Passive smoking means staying in a smoking environment for more than 15 min/week.

### 2.12. Inclusion and Exclusion Criteria

The following patients were included if they (1) met GDM diagnostic criteria during pregnancy 24-28 weeks, (2) were a singleton pregnancy, (3) were treated and delivered in the hospital, and (4) gave informed consent to the study and voluntarily signed informed consent.

The following patients were excluded: (1) had mental disease; (2) exclusion of GDM if venous fasting glucose value was <6.1 mmol/L and/or 2 h postload value of <7.8 mmol/L according to a previous literature [25]; (3) placenta previa, placenta abruption, pregnancy with history of recurrent abortion, or assisted pregnancies; and (4) received the treatment with other agents except of metformin, glyburide, and their analogues.

### 2.13. Patient Grouping

The study is a randomized, double-blind, and placebo-controlled trial. An allocation concealment was performed to hide the method of sorting trial participants into treatment groups. Strict diet control was applied to all participants, as follows: (1) a diet plan based on the patient's basic condition [26]; (2) monitoring of blood glucose; (3) calculation of the daily calorie requirement according to the patient's weight and making of an individual-compliant dietary plan to control dietary uptake; (4) advocacy of small meals and optimization of the dietary structure to ensure a balanced energy supply; and (5) scientific and rational exercise [27]. After middle pregnancy, 550 patients were determined with GDM and evenly assigned into the intervention group (received 500 mg cod-liver oil in capsule form daily) and control group (received placebo). The duration was from middle pregnancy to late pregnancy. Among them, the intervention group had an FBG of 6.67 ± 0.86 mmol/L. In the control group patients, the mean fasting blood glucose was 6.34 ± 0.98 mmol/L. There were no significant differences in age, gestational age, and fasting blood glucose levels between the two groups (*P* > 0.05).

### 2.14. Measurement of Primary Outcomes

Primary outcomes of the present study were the differences in the changes of metabolic parameters between the two groups during cod-liver oil consumption, including glycosylated hemoglobin A1c (HbA1c), fasting plasma glucose (FPG), 2 h plasma glucose (2hPG), and homeostatic model assessment for insulin resistance (HOMA-IR). At baseline and after 4-week randomization, blood samples were obtained after an overnight fast. HbA1c was measured by HPLC (model HLC-723 G7; Tosoh Corporation, Tokyo, Japan) using standard mode and by immunoassay (DCA 2000+ HbA1c cartridges, Siemens Corp., Tarrytown, NY, USA) [28]. FPG and 2hPG were measured by using the hexokinase method (ADVIA Autoanalyzer; Siemens Healthcare Diagnostics, Erlangen, Germany). HOMA-IR was measured by multiplying fasting plasma insulin (mU/L) and FPG (mmol/L) divided by 22.5 [29].

Blood lipid levels were detected in the early morning, 5 mL of the upper arm blood sample was collected on an empty stomach, and the supernatant was obtained by centrifugation at room temperature. Lipid profiles (triglyceride (TG), total cholesterol (TC), low-density lipoprotein cholesterol (LDL-C) ratio, and high-density lipoprotein cholesterol (HDL-C) ratio) were measured with a Beckman Au5800 automatic biochemical analyzer (Fullerton, CA, USA) via the corresponding assay kits from Abbott Laboratories (Abbott Park, IL, USA).

### 2.15. Measurement of Secondary Outcomes

Secondary outcomes include metabolic parameters and hs-CRP levels and measured between the two groups before and after 12-16 weeks of intervention from middle pregnancy to late pregnancy. FPG, 2hPG, lipid profiles, and HOMA-IR were measured according to the above method. hs-CRP was measured using an ELISA kit from Alpha Diagnostic Int. (San Antonio, Texas, USA) according to the manufacturer's instructions. Perinatal complications were observed between two groups, including as pregnancy-induced hypertension, polyhydramnios, premature delivery, postpartum hemorrhage, postpartum infection, premature rupture of membranes, and cesarean section. The women with hypertensive disorders were measured by using digital reactive hyperemia peripheral arterial tonometry and treated with aspirin [30]. Polyhydramnios were diagnosed with maximal vertical pocket (MVP) and treated with amniotic fluid decompression and indomethacin [31]. Premature delivery was observed and treated with *β*-adrenergic drugs and steroids or with cervical cerclage [32]. Postpartum hemorrhage was recorded by volume measurement and weight and treated with obstetrics and gynecology in an intensive care unit (ICU). Postpartum infection was estimated by using the surrogate outcome of use of postpartum antibiotics and treated with intravenous fluids and antibiotics [33]. Premature rupture of membranes was confirmed via ultrasound assessment and treated with an amniopatch [34]. Cesarean section was measured according to Cohen's kappa coefficient and treated with intramuscular methotrexate and bilateral uterine embolization [35].

### 2.16. Statistical Analysis

In this study, blood sugar level was the main variable of GDM and used to calculate sample size. The sample size was calculated as 500 by using Cochran's formula [36]. All data were presented as mean values ± S.D. (standard deviations) and mean differences (95% confidence interval, CI), as well as report OR (95% CI). The data were recorded by using EpiData3.1 software, and the questionnaire was provided immediately after actual food intake. The SAS9.4 software was used for data analysis. The measured data of the normal distribution was represented by the count data expressed as a percentage or rate. The count data were analyzed by chi-square (*χ*^2^). Univariate analysis of GDM-influencing factors was performed using *t*-test, and multivariate analysis was performed using unconditional logistic regression analysis. To explore the effects of sample size, Cohen's *d* was used to compare the outcomes from a continuous variable [37]. The baseline characteristics (age, BMI, and HbA1c) were adjusted between two groups to maintain that the variances of the two groups are the same. The magnitude of difference between the intervention and control groups was expressed in the difference between the means of the two groups. *P* < 0.05 was considered statistically significant.

## 3. Results

### 3.1. EPA and DHA Are the Main Components of Cod-Liver Oil

**Figure 1:**
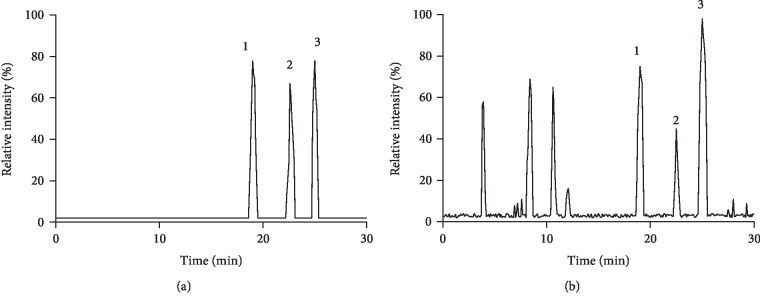
GC analysis of cod-liver oil: (a) standard solution; (b) sample solution; 1: EPA methyl ester; 2: C23:0 methyl ester; 3: DHA methyl ester.

GC analysis showed that the eluting time of EPA and DHA was at 18.9 min and 249.6 min, respectively (Figure 1(a)). EPA and DHA are the main components of cod-liver oil (Figure 1(b)), suggesting that cod-liver oil exerts its protective function for GDM mainly via EPA and DHA since they are the main components of omega-3, which was found to reduce the levels of FPG, HOMA-IR, and hs-CRP in the women with GDM [9].

### 3.2. Baseline Characteristics

During the therapy, 7 and 5 cases were lost in the intervention and control groups, respectively. Thus, 268 and 270 cases finished the whole experiment in the intervention and control groups, respectively. The age of pregnant women was 22.74-33.5 years old, with an average of 27.07 ± 5.58 years old. The education level is high school/secondary school/college degree, followed by university and above; the monthly income of the family was mostly at medium level, 2000-5000 RMB (Table 4). The statistical difference for all parameters was insignificant between the two groups (*P* > 0.05, Table 4). The dietary energy and main nutrient intakes of the pregnant women in the intervention group and the control group were statistically described in early pregnancy, middle pregnancy, and late pregnancy.  Table 5 showed that the statistical difference for all parameters was insignificant between the two groups (*P* > 0.05).

### 3.3. Primary Outcomes

The analysis of the mean differences (95% CI) for the primary outcome showed that the statistical differences for HbA1c, FPG, 2hPG, lipid profiles, and HOMA-IR were insignificant between the two groups (Table 6, *P* > 0.05).

### 3.4. Cod-Liver Oil Reduced the Levels of HbA1c and Plasma Glucose

There was no significant difference in blood glucose levels between the two groups before diet control (*P* > 0.05). After cod-liver oil consumption, the mean differences (95% CI) of HbA1c, FBG, and 2hBG in the intervention group were significantly lower than those in the control group (*P* < 0.05, Table 7). After adjustment for baseline characteristics, the statistical difference remained significant (*P* < 0.05, Table 7). The results suggested that cod-liver oil reduced plasma glucose.

### 3.5. Cod-Liver Oil Reduced the Levels of HOMA-IR and hs-CRP

Before treatment, there was no significant difference in the mean differences (95% CI) for the levels of hs-CRP and HOMA-IR between the two groups (*P* > 0.05, Table 8). The mean differences (95% CI) for the levels of hs-CRP and HOMA-IR of the patients in the intervention group were significantly lower than those in the control group (*P* < 0.05, Table 8). After adjustment for baseline characteristics, the statistical difference remained significant (*P* < 0.05, Table 8). The results suggested that cod-liver oil reduced the levels of HOMA-IR and hs-CRP.

### 3.6. Cod-Liver Oil Reduced Plasma Lipid Levels

There was no significant difference for the mean differences (95% CI) in the levels of blood lipid between the two groups before diet control (*P* > 0.05, Table 9). After cod-liver oil consumption, the levels of TG, TC, and LDL-C were reduced while no change for HDL-C in the intervention group when compared with the control group (*P* < 0.05, Table 9). The results suggested that cod-liver oil reduced plasma lipid levels.

### 3.7. Cod-Liver Oil Reduced Perinatal Complications of GDM Patients

After diet control, the OR (95% CI) for the incidence of perinatal complications such as pregnancy-induced hypertension, polyhydramnios, premature delivery, postpartum hemorrhage, postpartum infection, premature rupture of membranes, and cesarean section of the patients in the intervention group was significantly lower than that in the control group (*P* < 0.05, Table 10). The results suggested that cod-liver oil reduced perinatal complications of GDM patients.

## 4. Discussion

Cod-liver oil intervention effectively reduced the levels of blood glucose, hs-CRP, and HOMA-IR and the incidence of perinatal complications and improved pregnancy outcomes, suggesting that cod-liver oil has important clinical application value in the prevention of GDM. EPA and DHA are the main components of cod-liver oil, and thus, cod-liver oil may improve biochemical indices of GDM patients via EPA and DHA.

Repeated administration of DHA has been reported to decrease blood glucose, TAG, and NEFA levels and increase insulin sensitivity during insulin tolerance test and reduced adiposity [38]. EPA also contributed to weight loss and the decrease in the levels of blood glucose and total cholesterol [39]. The results are consistent with our findings that the consumption of cod liver with rich DHA and EPA reduced the levels of blood glucose and HOMA-IR (Tables 7 and 8). The results suggest that cod liver possibly controls blood glucose levels and insulin resistance of GDM patients via its main components DHA and EPA. On the other hand, although most patients had taken metformin, glyburide, and their analogues, the blood glucose was still high before the recruitment. The addition of cod-liver oil may improve the resistance of metformin, glyburide, and their analogues.

The addition of EPA to diet was found to reduce the levels of hs-CRP and prevent inflammatory response [40]. DHA was also observed to suppress the inflammatory factor such as hs-CRP by altering blood lipids and their fatty acid composition [41]. The results are also consistent with our findings that the consumption of cod liver with rich DHA and EPA reduced the levels of hs-CRP (Table 8). The results suggest that cod liver possibly controls inflammatory levels of GDM patients via its main components DHA and EPA. DHA and EPA also affect plasma lipid parameters (Table 9), oxidative, and fatty acid composition [12], which can further improve inflammation situation [42, 43].

EPA and DHA have been widely reported to have protective functions for GDM patients [44–46]. However, EPA and DHA were electrochemically inactive and cannot be measured directly. In this study, the catalytic efficiency of boron/sulfuric acid-methanol solution was the same, and the methanol solution of hydrochloric acid was obviously lower than the former two. It has been proved by method that the methanol solution of sulfuric acid can completely replace the boron trifluoride methanol solution, which can not only effectively reduce the detection cost but also meet the requirements of green chemistry. At the same time, it was found that the methanol solution of sulfuric acid was used as the catalyst, the supernatant clarification time was significantly shorter than that of the boron trifluoride catalyst, and the supernatant liquid was quickly obtained, the operation time was shortened, and the efficiency was improved.

The saponification and methylation conditions were selected; the saponification time was 5, 10, 20, and 30 min; the esterification time was 5, 10, 15, and 20 min; and the saponification/methylation temperature was 70, 80, 90, and 100°C. Taking EPA and DHA contents as indicators, the results show that the precolumn derivatization conditions of the test products were optimized in the best conditions. The contents of EPA and DHA were high, and the method was the stable for detecting the omega-3 contents in cod-liver oil. The calibration factor was used to determine the content of specific fatty acids EPA and DHA in cod-liver oil. The method was simple, accurate, reproducible, and precise. It could be used for the quality identification of cod-liver oil.

Gestational diabetes is the first type of endocrine disease with abnormal glucose metabolism during pregnancy. Most of the postpartum can return to normal, but the chance of developing type 2 diabetes is greatly increased. The poor blood sugar control during pregnancy is also prone to polyhydramnios [47] and pregnancy-induced hypertension [48, 49]. The main pregnancy complications include late preterm birth [50], hyperlipidemia [51], and posttraumatic stress disorder and antepartum complications [52]. The basic pathogenesis of gestational diabetes is related to insulin resistance. The increase of secretion of various placental hormones such as prolactin, estrogen, and progesterone during pregnancy can cause physiologically insufficient or absolute secretion of insulin [53], and mild insulin resistance is widespread in pregnant women, but some pathological factors can cause GDM caused by glucose metabolism disorder in pregnant women [54]. The relationship between inflammatory factors and adipocytokines in insulin resistance was close in pregnant women [55].

The study of the mechanism of insulin resistance in pregnant women provided a new idea for the prevention and treatment of clinical gestational diabetes with great clinical significance. GDM patients are positively correlated with CRP [56, 57] and HOMA-IR [58]. In recent years, exercise therapy [59, 60] and diet therapy have been gradually applied to the treatment of GDM [61, 62], and diet therapy is the most basic treatment of GDM. Individualized diet control is to use scientific and reasonable diet to control the progress of the disease and prevent poor prognosis. The patients with simple diet and moderate exercise can achieve satisfactory glycemic control [63, 64]. The purpose of diet therapy for GDM patients is to control the blood glucose to the desired level while meeting the essential nutrients of pregnant women and fetuses, to avoid the occurrence of hunger ketoacidosis in pregnant women and to reduce the incidence of complications. In this study, cod-liver oil control FPG, 2hPG, hs-CRP, and HOMA-IR had significant effects (*P* < 0.05). In addition, diet control was also found to reduce pregnancy-induced hypertension and polyhydramnios in patients in an intervention group. The perinatal complications included premature delivery, postpartum hemorrhage, and postpartum infection, and their incidence in the intervention group was lower than that in the placebo group (*P* < 0.05). This shows that in the clinical treatment of GDM patients, the implementation of cod-liver oil intervention can control blood glucose levels, while improving pregnancy outcomes and reducing perinatal complications.

There were some limitations in the present study. Serum DHA and EPA will reflect the actual result of cod-liver oil consumption. Unfortunately, maternal serum levels of DHA and EPA were not measured because of the shortage of the study design. Further work is needed to address these important issues, although cod-liver oil consumption improved metabolic indices and hs-CRP levels in GDM patients via DHA and or EPA. However, the exact molecular mechanism of the functional food remains unclear. We require further work to address these issues in the future.

## 5. Conclusions

In sum, in the clinical treatment of GDM patients, cod-liver oil intervention can effectively control the patient's blood glucose and lipid levels and can reduce the incidence of perinatal complications and improve pregnancy outcomes and has important clinical application value, of worthy in-depth promotion.

## Figures and Tables

**Table 1:** Relative correction factor.

Components	*F*_*X*_			$\bar{FX}$			RSD (%)	Total $\bar{FX}$
EPA	1.023	1.022	1.021	1.024	1.018	1.022	0.23	1.022
	1.018	1.022	1.027	1.025	1.025	1.023	0.34	

DHA	1.037	1.033	1.034	1.035	1.030	1.034	0.25	1.034
	1.030	1.032	1.038	1.035	1.035	1.034	0.30	

Note: calculation formula: *F*_*X*_ = *A*_*S*_ · *W*_*X*_/(*A*_*X*_ · *W*_*S*_) · *A*_*X*_: peak area of EPA methyl ester or DHA methyl ester; *A*_*S*_: peak area of C23:0 methyl ester; *W*_*S*_, C23:0 methyl ester addition (mg); *W*_*X*_: EPA methyl ester or DHA methyl ester addition (mg).

**Table 2:** Recovery rates of EPA and DHA.

	Sample (mg)	Content (mg)	Added standards (mg)	Measured content	Recovery rate (%)	Average recovery rate (%)	RSD (%)
EPA	49.65	3.428	2.829	6.182	97.33	98.7	0.98
	51.11	3.529	2.829	6.342	99.46		
	49.72	3.433	2.829	6.238	99.16		
	51.07	3.508	3.536	7.022	99.38		
	51.89	3.583	3.536	7.062	98.38		
	51.98	3.589	3.536	7.141	100.43		
	48.25	3.338	4.244	7.523	98.60		
	49.01	3.422	4.244	7.574	97.84		
	48.55	3.352	4.244	7.509	97.95		

DHA	50.51	6.885	5.783	12.563	98.18	99.4	1.34
	51.11	7.087	5.783	12.891	100.35		
	49.72	6.894	5.783	12.678	100.00		
	51.07	7.081	7.229	14.309	99.97		
	51.89	7.196	7.229	14.249	100.05		
	51.98	7.208	7.229	14.570	101.83		
	48.25	6.705	8.675	15.257	98.59		
	49.01	6.871	8.675	15.342	97.66		
	48.55	6.733	8.675	15.261	98.31		

**Table 3:** EPA and DHA content in cod-liver oil.

Patch no.	EPA (mg/mL)	DHA (mg/mL)
170411	76.08	151.65
170420	73.42	145.95
170508	74.75	148.30
170513	76.24	147.60
170518	75.66	150.76
170524	74.03	149.34

**Table 4:** Baseline characteristics between two groups, *n*(%).

Parameters	Intervention group (*n* = 268)	Control group (*n* = 270)	*t* or *χ*^2^ values	*P* values
Age (years)	28.12 ± 5.38	27.29 ± 6.02	0.412	0.544
Educational level				
Junior high school and below	27(10.07)	31(11.48)		
High school/secondary school/college	162(60.45)	156(57.78)	0.480	0.786
University and above	79(29.48)	83(30.74)		
Monthly income (RMB)				
<2000	32(11.94)	29(10.74)		
2000-5000	201(75)	194(71.85)	1.522	0.467
>5000	35(13.06)	45(16.67)		
Place of residence				
Rural	72(26.87)	85(31.48)	1.387	0.239
City	196(73.13)	185(68.52)		
Sports activities (h/d)				
<7	53(19.78)	62(22.96)		
7-10	162(60.45)	156(57.78)	0.820	0.664
>10	53(19.78)	52(19.26)		
Passive smoking	32(11.94)	36(13.33)	0.595	0.440
Primitive	18(6.72)	22(8.15)	0.401	0.527
Family history of diabetes	12(4.48)	10(3.7)	0.205	0.650
Family history of hypertension	41(15.3)	37(13.7)	0.276	0.599
Hypertension during pregnancy	53(19.78)	59(21.85)	0.352	0.553
Prepregnancy BMI (kg/m^2^)	23.41 ± 4.29	24.17 ± 3.16	0.248	0.624
Oral medications				
Metformin	87(32.46)	90(33.33)	0.182	0.980
Glyburide	65(24.25)	68(25.19)		
Analogues	116(43.28)	112(41.48)		
Weight gain during pregnancy				
Insufficient	46(17.16)	42(15.56)	2.056	0.358
Appropriate	180(67.16)	173(64.07)		
Excessive	42(15.67)	55(20.37)		

Note: *n* = 268 in the interventional group and *n* = 270 in the control group.

**Table 5:** Dietary energy and main nutrient intake of pregnant women in different periods.

Parameters	Intervention group	Control group	*t* values	*P* values
Energy intake (kcal/d)				
Early pregnancy	1347.40 ± 464.60	1348.50 ± 457.70		
Middle pregnancy	1387.70 ± 471.60	1382.70 ± 462.00	0.160	0.797
Late pregnancy	1403.20 ± 464.10	1411.50 ± 459.50		
Protein intake (g/d)				
Early pregnancy	60.72 ± 21.48	60.56 ± 20.56		
Middle pregnancy	62.07 ± 21.66	61.75 ± 20.86	0.115	0.804
Late pregnancy	62.68 ± 21.35	62.92 ± 21.06		
Fat intake (g/d)				
Early pregnancy	42.31 ± 23.38	41.46 ± 23.28		
Middle pregnancy	43.73 ± 23.22	42.75 ± 32.54		
Late pregnancy	43.85 ± 22.79	43.22 ± 23.83	0.266	0.614
Total fatty acid intake (g/d)				
Early pregnancy	22.90 ± 18.22	22.39 ± 18.92		
Middle pregnancy	23.18 ± 17.97	22.66 ± 19.04	0.099	0.809
Late pregnancy	23.14 ± 17.12	22.80 ± 19.10		
Saturated fatty acid intake (g/d)				
Early pregnancy	6.75 ± 4.54	6.63 ± 4.70		
Middle pregnancy	6.84 ± 4.48	6.71 ± 4.74	0.154	0.798
Late pregnancy	6.84 ± 4.14	6.77 ± 4.76		
Monounsaturated fatty acid intake (g/d)				
Early pregnancy	8.69 ± 6.97	8.49 ± 7.23		
Middle pregnancy	8.79 ± 6.87	8.58 ± 7.28	0.225	0.714
Late pregnancy	8.77 ± 6.78	8.64 ± 7.30		
Polyunsaturated fatty acid intake (g/d)				
Early pregnancy	7.30 ± 6.65	7.12 ± 6.92		
Middle pregnancy	7.40 ± 6.57	7.20 ± 6.96	0.189	0.738
Late pregnancy	7.37 ± 6.48	7.24 ± 6.98		
Carbohydrate intake (g/d)				
Early pregnancy	198.60 ± 78.33	196.10 ± 70.59		
Middle pregnancy	203.10 ± 71.52	201.70 ± 72.65	0.075	0.888
Late pregnancy	208.30 ± 71.36	204.80 ± 72.92		
Dietary fiber intake (g/d)				
Early pregnancy	29.04 ± 9.19	29.47 ± 9.65		
Middle pregnancy	29.34 ± 9.44	29.66 ± 9.71	0.040	0.931
Late pregnancy	29.47 ± 9.48	30.00 ± 9.66		
Soluble dietary fiber intake (g/d)				
Early pregnancy	1.91 ± 2.07	1.87 ± 2.17		
Middle pregnancy	1.93 ± 2.05	1.89 ± 2.20	0.099	0.809
Late pregnancy	1.92 ± 2.03	1.89 ± 2.19		
Insoluble dietary fiber intake (g/d)				
Early pregnancy	5.17 ± 4.95	5.10 ± 5.22		
Middle pregnancy	5.21 ± 4.90	5.16 ± 5.41	0.004	0.957
Late pregnancy	5.17 ± 4.83	5.15 ± 5.28		
Cholesterol intake (mg/d)				
Early pregnancy	60.54 ± 58.29	56.76 ± 52.66		
Middle pregnancy	59.75 ± 58.35	55.41 ± 52.50	0.266	0.614
Late pregnancy	60.61 ± 60.28	57.07 ± 55.00		

Note: *n* = 268 in the interventional group and *n* = 270 in the control group. The statistical difference was significant if *P* < 0.05.

**Table 6:** The comparison of primary outcome between two groups.

	Control group, MD (95% CI)	Intervention group, MD (95% CI)	*t* values	*P* values
Fasting plasma glucose (mmol/L)	8.13 (7.72, 8.61)	8.37 (8.07, 9.15)	0.044	0.731
2 h postprandial plasma glucose (mmol/L)	9.85 (9.34, 10.58)	9.53 (9.17, 10.29)	0.126	0.697
HOMA-IR	3.37 (3.21, 3.65)	3.31 (3.09, 3.84)	0.104	0.632

Note: HOMA-IR: homeostatic model assessment insulin resistance. *n* = 268 in the interventional group and *n* = 270 in the control group. Primary outcomes were measured after 4-week randomization. MD: mean differences; CI: confidence interval. The difference between the intervention group and the control group was statistically significant if *P* < 0.05.

**Table 7:** The comparison of blood glucose between two groups before and after adjusting for confounders (age, BMI, and HbA1c).

Groups	HbA1c (%), MD (95% CI)		FBG (mM), MD (95% CI)		2hBG (mM), MD (95% CI)	
	Before therapy	After therapy	Before therapy	After therapy	Before therapy	After therapy
Before adjustment						
Control	6.40 (6.17, 6.58)	6.49 (6.18, 6.61)	8.31 (7.95, 8.74)	6.37 (6.13, 6.84)	9.75 (8.91, 10.96)	7.34 (6.41, 8.65)
Intervention	6.35 (6.12, 6.50)	5.82 (5.51, 6.08)	8.93 (8.14, 9.76)	5.28 (5.09, 5.54)	10.25 (8.86, 11.14)	5.68 (5.19, 6.26)
*t* values	0.513	3.587	0.237	3.982	0.169	5.896
*P* values	0.384	0.022	0.621	0.013	0.785	0.001
After adjustment						
Control	6.20 (6.14, 6.40)	6.15 (5.99, 6.37)	8.13 (7.82, 8.49)	6.01 (5.75, 6.39)	9.47 (8.39, 10.36)	7.55 (6.28, 8.12)
Intervention	6.27 (6.03, 6.41)	5.43 (5.21, 5.82)	8.24 (8.01, 8.59)	5.17 (4.93, 5.30)	9.33 (8.15, 10.01)	5.62 (5.03, 6.07)
*t* values	0.132	4.825	0.245	4.692	0.169	6.712
*P* values	0.689	0.012	0.734	0.006	0.793	0.001

Note: *n* = 268 in the interventional group and *n* = 270 in the control group. The therapy duration was the whole stage of pregnancy. MD: mean differences; CI: confidence interval. The difference between the intervention group and the control group was statistically significant if *P* < 0.05.

**Table 8:** The comparison of hs-CRP and HOMA-IR between two groups before and after adjusting for confounders (age, BMI, and HbA1c).

Groups	hs-CRP (mmol/L), MD (95% CI)		HOMA-IR, MD (95% CI)	
	Before therapy	After therapy	Before therapy	After therapy
Before adjustment				
Control	26.87 (21.05, 31.92)	23.45 (22.53, 24.81)	3.41 (3.12, 3.69)	3.84 (3.07, 4.29)
Intervention	27.16 (22.14, 32.47)	9.31 (6.74, 11.54)	3.27 (2.83, 4.15)	1.85 (1.19, 2.44)
*t* values	0.335	14.263	0.253	8.986
*P* values	0.269	0.001	0.512	0.001
After adjustment				
Control	25.99 (21.14, 31.82)	22.81 (22.07, 23.95)	3.07 (2.92, 3.16)	2.93 (1.92, 3.86)
Intervention	26.31 (21.52, 33.18)	8.44 (6.34, 10.27)	3.15 (2.64, 3.85)	1.74 (1.26, 2.31)
*t* values	0.458	15.616	0.375	9.321
*P* values	0.312	0.001	0.414	0.001

Note: *n* = 268 in the interventional group and *n* = 270 in the control group. The therapy duration was the whole stage of pregnancy. MD: mean differences; CI: confidence interval. The difference between the intervention group and the control group was statistically significant if *P* < 0.05.

**Table 9:** The comparison of lipid profiles between intervention and control groups.

	TG, MD (95% CI)	TC, MD (95% CI)	HDL-C, MD (95% CI)	LDL-C, MD (95% CI)
Before therapy				
Intervention	1.65 (1.13, 2.35)	4.47 (3.98, 5.95)	1.35 (1.24, 1.50)	3.01 (2.74, 3.29)
Control	1.58 (1.26, 2.01)	4.86 (3.99, 5.99)	1.41 (1.20, 1.59)	3.12 (2.71, 3.48)
*t* value	0.652	0.548	0.241	0.187
*P* value	0.413	0.473	0.698	0.713
After therapy				
Intervention	1.46 (0.85, 2.17)	4.17 (3.32, 5.30)	1.28 (1.15, 1.47)	2.44 (2.13, 2.61)
Control	1.68 (1.24, 2.10)	4.83 (3.71, 6.01)	1.34 (1.11, 1.52)	3.10 (2.68, 3.37)
*t* value	4.981	4.364	0.684	9.135
*P* value	0.021^∗^	0.019^∗^	0.125	0.002^∗^

Note: MD: mean differences; CI: confidence interval. ^∗^*P* < 0.05 vs. a control group.

**Table 10:** Comparison of perinatal complications between the two groups, cases (%).

	Control, OR (95% CI)	Intervention, OR (95% CI)	*t* values	*P* values
Pregnancy-induced hypertension	0.98 (0.53, 1.48)	1.03 (0.68, 1.65)	0.684	0.321
Amniotic fluid	1.24 (0.85, 4.12)	1.14 (0.71, 2.82)	0.893	0.197
Premature delivery	1.35 (0.73, 5.24)	1.26 (0.53, 3.17)	0.541	0.537
Postpartum hemorrhage	1.06 (0.43, 1.98)	1.01 (0.66, 1.69)	0.142	0.735
Postpartum infection	2.18 (0.67, 9.44)	1.02 (0.21, 1.79)	8.961	0.003
Premature rupture of membranes	0.91 (0.67, 1.38)	0.99 (0.43, 2.11)	0.819	0.135
Cesarean section	1.54 (0.89, 3.76)	1.16 (0.76, 1.98)	8.267	0.004
Incidence rate (%)	9.22 (1.98, 27.4)	7.61 (3.3, 13.47)	19.356	0.000

Note: *n* = 268 in the interventional group and *n* = 270 in the control group. The therapy duration was the whole stage of pregnancy. OR: odds ratio; CI: confidence interval. The difference between the intervention group and the control group was statistically significant if *P* < 0.05.

## Data Availability

The data for the current study are available from the corresponding author upon reasonable request.
